# Effectiveness of home fire safety interventions. A systematic review and meta-analysis

**DOI:** 10.1371/journal.pone.0215724

**Published:** 2019-05-20

**Authors:** Maya Senthilkumaran, Goris Nazari, Joy C. MacDermid, Karen Roche, Kim Sopko

**Affiliations:** 1 School of Rehabilitation Science, McMaster University, Hamilton, ON, Canada; 2 School of Physical Therapy, Health and Rehabilitation Science, Western University, London, ON, Canada; 3 Collaborative Program in Musculoskeletal Health Research, Bone and Joint Institute, Western University, London, ON, Canada; 4 Roth McFarlane Hand and Upper Limb Centre, St. Joseph’s Hospital, London, ON, Canada; 5 Burlington Fire Department, Burlington, ON, Canada; University of Mississippi Medical Center, UNITED STATES

## Abstract

**Purpose:**

To assess the effectiveness of Home Fire Safety (HFS) interventions versus other interventions/no interventions/controls on HFS knowledge and behaviour at short-, intermediate- and long-term follow ups.

**Design:**

Systematic review and meta-analysis of randomized controlled trials.

**Data sources:**

MEDLINE, EMBASE and PubMed databases were searched from January 1998 to July 2018, and studies retrieved.

**Participants:**

Toddlers, children (primary or secondary school), teenagers or adults.

**Interventions/Comparison:**

HFS interventions compared to other interventions / no interventions / controls.

**Outcomes:**

HFS knowledge and behaviour.

**Results:**

10 studies were identified (8 RCTs and 2 prospective cohort). Two studies assessed the effects of HFS interventions vs no interventions on HFS knowledge at up to 4 months follow up in school children and demonstrated significant difference between groups (very low quality, 2 RCTs, 535 participants, SMD 0.38, 95% CI: 0.21 to 0.55, p < 0.001). One study examined the effects of different modes of HFS interventions (computer-based vs instructor-led) on HFS knowledge and behaviour immediately post-intervention in adults and displayed no significant difference between groups (HFS knowledge; very low quality, 1 RCT, 68 participants, SMD -0.02, 95% CI: -0.50 to 0.45, p = 0.92) and (HFS behaviour; very low quality, 1 RCT, 68 participants, SMD 0.06, 95% CI: -0.41 to 0.54, p = 0.79) respectively.

**Conclusion:**

The limited evidence supports the use of HFS interventions to improve HFS knowledge and behaviour in children, families with children and adults.

## Introduction

Every day, 7 people die from home fires in the United States (US) [1]. Residential fires remain a major public health burden [2–4]. Between 2011 and 2015, U.S. fire departments responded to an average of 358,500 home structure fires per year, which resulted in an average of 2,510 fatalities annually [1]. In 2016, the rate at which U.S. home structure fires were reported was 1.1 per thousand population [1]. In United Kingdom, it was estimated that at least 500 deaths and 15,000 injuries were due to residential fires in 1998 [5–6].

Fire prevention requires multiple strategies. One strategy is to identify and target risk factors. Several systematic reviews have identified factors associated with higher rates of fires high number of residents, male homeowner, children under age of 5 years, smoking, low-income, buildings in poor conditions, frailty/disability, young and old age tenants, as the distinguishing risk factors associated with such incidents [7–9].

Another approach to fire safety is early detection of fire initiation in the homes, to prevent progression. To date, two meta-analyses examined smoke alarm coverage interventions by comparing the intervention to no interventions or to usual care [10–11]. A later network meta-analysis evaluated the effectiveness of smoke alarm interventions, but instead, compared several types of interventions, and found that the most effective method was the most intensive (includes education, low cost equipment fitting and in-home safety inspection) [12]. Home Fire Safety (HFS) knowledge and behaviour outcomes were also examined in a 1999 review [13]. The results concluded that there is a need for program evaluation especially among school-based education programs.

While the review by Warda et al. (1999) provides valuable insights, it has important limitations. For example, the review is outdated, included no critical appraisal or meta-analyses. Furthermore, since 1999, numerous studies have emerged [14–27], which warrants the need for a systematic review and meta-analysis. Therefore, the aims of this review were:

1. to quantify the effects of Home Fire Safety (HFS) interventions versus other interventions/no interventions/controls on HFS knowledge and behaviour at short-, intermediate- and long-term follow ups,
2. to rate the quality of the body of literature that compares the effectiveness of HFS interventions versus other interventions/control according to GRADE guidelines across each outcome.

## Methods

We followed the Preferred Reporting Items for Systematic Reviews and Meta-Analyses (PRISMA) and Cochrane collaboration guidelines [28–29] (S1 Checklist) PROSPERO registration number: CRD42018106866.

### Eligibility criteria

Studies were included in this systematic review if the below criteria were met [30–33]:

- *Design*: randomized controlled trials (RCTs) or non-randomized studies published in a peer reviewed journal,
- *Participants*: toddlers, children (primary or secondary school), teenagers or adults–no age limit,
- *Intervention/Comparison*: studies that compared HFS interventions to other interventions/no interventions/controls
- *Outcomes*: HFS knowledge and behaviour,

Reports, conference abstract and posters were excluded from this systematic review [30–33].

### Information sources

We conducted systematic electronic searches to identify relevant studies in MEDLINE, EMBASE and PubMed from January 1998 to July 2018. Several different combinations of keywords were used, such as: “home fire safety”, “home fire safety knowledge”, “home fire safety behavior”, “effectiveness of home”, fire safety, “residential fires”, “fire prevention programs”, “fire prevention programs adults”, “fire prevention programs children”, “fire prevention”. (S1 File). In addition, we carried out a manual search of the reference lists of the identified studies.

### Study selection

Two independent reviewers (MS and GN) performed the systematic electronic searches in each database. We then identified and removed the duplicate studies. In the next stage, we independently screened the titles and abstracts and retrieved in full text any article marked include or uncertain by either reviewer. Lastly, we carried out an independent full text review to assess final eligibility. In case of disagreement, a third reviewer, (JM), provided a consensus through discussion.

### Data collection process

Two independent researchers (MS and GN) extracted the data from the eligible studies. In case of disagreement, a third reviewer (JM), provided a consensus through discussion. Data extraction included the author, year, study setting, study population, sample size, age, intervention/comparison groups, follow up periods and the primary and secondary outcomes. When insufficient data were presented, GN contacted the authors by email and requested further data.

### Assessment of risk of bias in individual studies

Two independent review authors (JM and GN) assessed the RCTs and non-randomized studies for risk of bias. The risk of bias assessment in the included RCTs was performed using the Cochrane Risk of Bias tool [29]. The Cochrane Risk of Bias tool is based on 7 items, random sequence generation, allocation concealment, blinding of participants and personnel, blinding of outcome assessment, incomplete outcome data, selective reporting and other bias [29]. We defined the other bias category as trials that did not include statements on sources of funding/potential sources of conflicts of interest. The adequacy of each of the seven risk of bias domains was rated as “low”, “unclear” or “high” risk according to criteria provided in the Cochrane Handbook for Systematic Reviews of Interventions [29].

### Assessing the quality of evidence

We used the GRADE approach for systematic reviews, to determine the quality of evidence related to each outcome to summarize the extent of our confidence in the estimates of the effect [31–36]. The GRADE approach considers the risk of bias, publication bias, consistency of findings, precision, and the applicability of the overall body of literature to provide a rating of quality of evidence (high, moderate, low, or very low) per outcome [34–39].

### Summary measures

To quantify and interpret our data, a Minimal Clinically Important Difference (MCID) of 0.5 standard deviation points for HFS knowledge and behaviour was used [40]. Timing of outcome assessment were categorised as short-term (3–4 months), intermediate-term (6 months) and long-term (12 months).

### Subgroup analysis and exploring heterogeneity

In the presence of heterogeneity, we planned to perform the following subgroup analyses (a priori): trials at low risk of bias (low risk of bias in allocation concealment and blinding of outcome assessor), type of HFS intervention used. An I^2^ estimate of at least 50% and a statistically significant Chi^2^ statistic (P = 0.10) were used to indicate evidence of a substantial problem with heterogeneity [41].

### Synthesis of results

We performed 7 meta-analyses of studies comparing HFS interventions to other interventions/no interventions/controls, using the outcomes HFS knowledge or behaviour, at short-, intermediate- and long-term follow ups. We used the Review Manager 5.3 (RevMan 5.3) software to conduct our review and used the standardized mean difference (SMD) with a random-effects model to pool outcomes.

## Results

### Study selection

**Fig 1:**
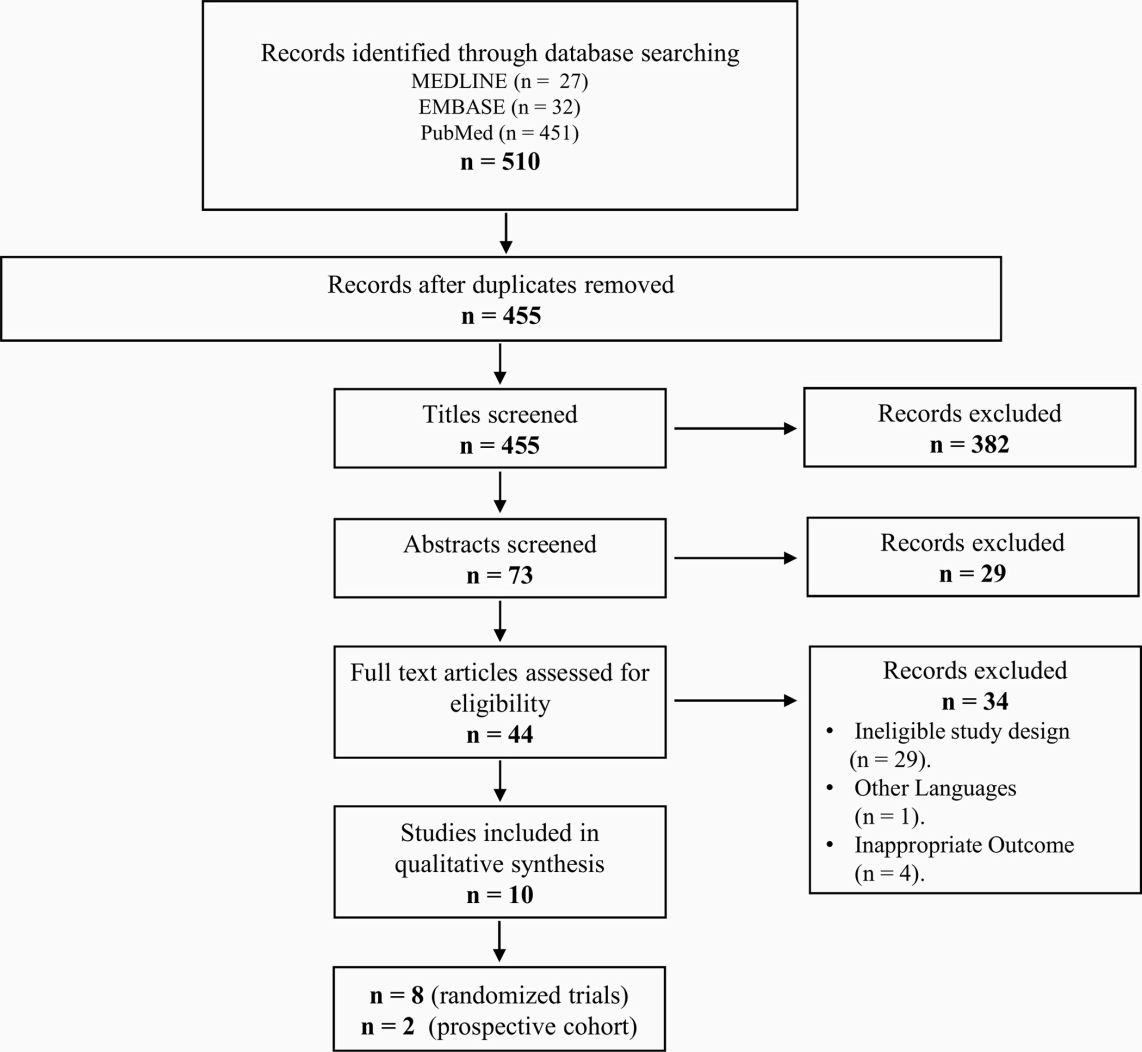
Selection of studies for inclusion in the systematic review.

Initially, our search identified 510 publications. After removal of the duplicates, 455 articles remained and were screened using their title and abstract, leaving 44 studies for full text review. Of these, 10 studies were eligible (8 RCTs and 2 prospective cohort) [18–27]. The flow of studies through the selection process is presented in Fig 1.

### Study characteristics

The 8 eligible RCTs were conducted between 2003–2017 and included 1962 participants. Study size ranged from 76 to 499 participants. Trials were conducted in Canada, USA and UK [18–20, 22–26]. Only two out of the eight trials were registered in a clinical trials register [20,22]. In addition, three trials did not include statements on sources of funding or potential sources of conflicts of interest [24–26]. A summary description of all the included RCTs is displayed in Table 1. The 2 eligible prospective cohort studies were conducted in 2003 and 2017, and included 1491 participants (study sizes were 671 and 820). Studies were conducted in UK and Australia. A summary description of all the included studies is displayed in Table 2.

**Table 1:** Study characteristics of the included randomized controlled trials.

Study	Population	Outcomes	Follow-ups	Intervention/Comparison
**Lehna et al. (2014)** Louisville, Kentucky, USA	Parents of children with and without special needs: n = 87 School-Based:40 (special needs: 12.3±1.8 yrs., without: 11.2±0.8 yrs.) Waiting-Room:47 (special needs: 12.8±2.1 yrs., without: 11.1±1.2 yrs.)	-Home Fire Safety knowledge. -Home Fire Safety behaviour.	2 weeks	School-Based group: was shown a *Home Safe Home* DVD video. Waiting-Room group: was taught the same intervention but in a face-to-face manner.
**Hwang et al. (2006)** Philadelphia, Pennsylvania, USA	179 third and fourth grade students from a high-risk, poor, and minority tract. Intervention group:78 Control group:72	-Home Fire Safety knowledge. -Home Fire Safety behaviour.	4 weeks	Control Group: consisted of students who participated in the baseline and follow up surveys only. The students were administered the curriculum *Risk Watch* by the NFPA at the completion of the study. Intervention Group: same as the control group, with the addition to an in-home visit by fire department personnel. During the intervention, smoke alarms were installed, and a fire escape plan was developed on an erase board.
**Morrongiello et al. (2012)** Thunder Bay Ontario, University of Guelph and University of Alabama-Birmingham	76 children eligible for intervention Age:3.5 to 6 yrs., Mean Age:4.76±0.91yrs. Intervention: *The Great Escape*: n = 38. 53% boys, Mean age:4.77±0.96 Control: *The Blue Dog* n = 38, 47% boys Mean age:4.76±0.86	-Home Fire Safety knowledge.	3 weeks	Intervention (Great escape CD version): cartoon character, different hazard scenarios, children received corrective feedback. Control (Blue Dog CD version). cartoon character, different hazard scenarios, children received corrective feedback (except the focus was on how to behave safety near dogs).
**Harrington et al. (2003)** North Carolina USA	289 nursing staff. Instructor-led group (n = 137) Computer-based group (n = 152)	-Home Fire Safety knowledge. -Home Fire Safety behaviour.	Immediately	Computer-Based: Consisted of narration, interaction, animations, and engaging videos. The program was also designed to have move forward/backward features so that the learner could go on their own pace. Instructor-Led: Program curriculum was the same content as computer-based, just taught in a different method. The training was face-to-face and included manuals and videotapes.
**Deave et al. (2017)** Nottingham Bristol, Norwich and Newcastle England	n = 499 Injury Prevention Briefing+facilitation n = 241 Control n = 258	-Home Fire Safety knowledge. -Home Fire Safety behaviour.	12 months	Intervention (IPB + Facilitation): Included the IPB with the additional facilitation. The facilitation consisted of 3 follow ups via telephone or face-to-face. The research team evaluated use of home fire safety, quality of program, and smoke alarm coverage. Control: Usual fire prevention activity
**Kendrick et al. (2007)** Nottingham, UK	Primary schools were randomized. Children ranged from ages 7–10. Intervention arm:11 schools, 240 participants at baseline,203 participants at follow-ups. Control Arm:9 schools, 219 participants at baseline,188 participants at follow-ups.	-Home Fire Safety knowledge. -Home Fire Safety behaviour.	4 months	Intervention (Risk Watch): Curriculum was designed for teachers to educate their students about injury prevention such as falls, fire and burns, poisoning and bike safety. Teachers were trained by Fire Service personnel to teach to young audiences. 9 schools taught fire and burn prevention. Control (no intervention): control group received no intervention.
**Wang et al (2016)** Maryland, USA	Low-income families with toddlers. Total of 277 mother-toddler dyads. Safety intervention:(n = 91) Attention control:(n = 186)	- Home Fire Safety behaviour	6 and 12 months	Safety Intervention: Intervention covered fire prevention, fall prevention, poison control, and car seat use. It was delivered in two sites; church and preschool. Intervention was led by health educators. Attention-control: Intervention similar to safety, but was regarding maternal diet/physical activity or toddler feeding behaviour.
**Posner et al. (2003)** Philadelphia, Pennsylvania, USA	96 caregivers of children 5 or younger in an urban emergency department. Control Group: (n = 47), Age of caregiver: 30.7±8.8, Age of child:2.0±1.3 Intervention Group:(n = 49), Age of caregiver:27.6±6.4, Age of child:2.4±1.4	-Home Fire Safety knowledge.	~2 months	Intervention: received home safety counselling through verbal review and were given a home fire safety kit. Control: participants only received prevention information about their child’s type of injuries.

**Table 2:** Study characteristics of the included prospective cohort studies.

Study	Population	Outcomes	Follow-ups	Intervention/Comparison
**Lamb et al. (2006)** Bristol, UK	The study tested the effectiveness of a Life-skills program by randomly selecting children in schools for the intervention or control. The control group did not receive any intervention. (n = 671) 345 boys and 326 girls Girls:47% intervention,52% control	Home Fire Safety knowledge	Immediate, and 3 months	Life-skills Protocol: participants received a set of detailed skill programs. Control: control group received no intervention.
**Muller et al. (2013)** Queensland Australia	The trial was conducted in two regions within Queensland. One region received the intervention, the other region did not receive any intervention (control). Intervention was targeted towards adult burn prevention. Intervention Region (IR):(n = 405), Age:53±18 Control Region (CR):Pre-intervention:(n = 415), Age:54±18	Home Fire Safety knowledge	12 months	Intervention Region (IR): Participants in IR were given a multimedia intervention based on the theme, Don’t *be a flamin’ fool*. Medias included several television commercials educated audiences about burns and first-hand experiences about being a victim. Control Region (CR): participants in the CR received no intervention.

### Risk of bias assessment in the individual studies

**Fig 2:**
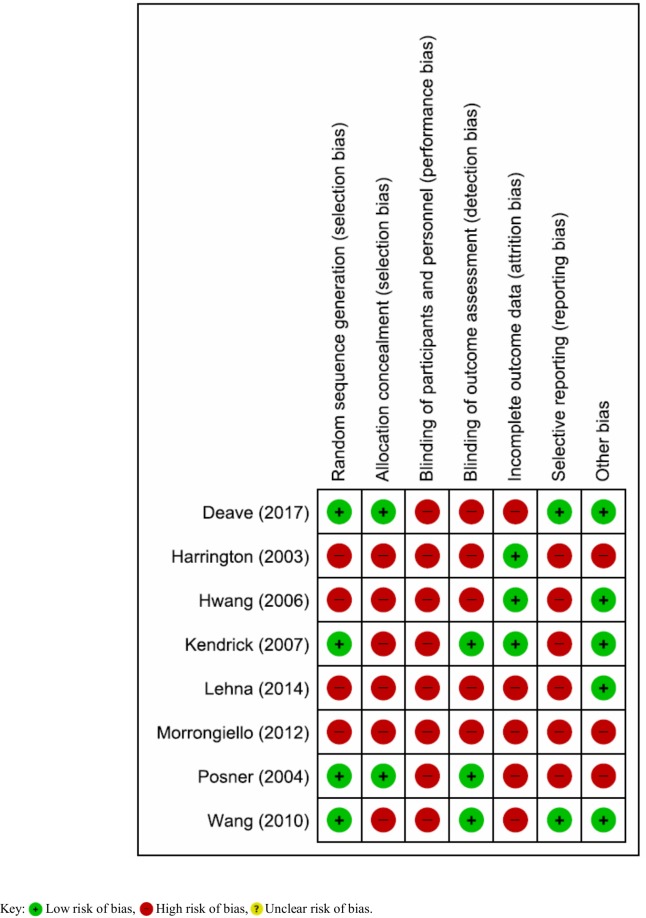
Risk of bias summary: Review authors’ judgements about each risk of bias item for each included study.

The risk of bias assessment is presented in Fig 2. Performance bias (lack of or inadequate blinding of participants who could influence how interventions, including co-interventions are performed/administered) was rated at high risk in all the included trials (n = 8) [18–20, 22–26]. Detection bias [18, 22–25] (lack of or inadequate blinding of participants who could influence the measurement or interpretation of outcomes) and attrition bias [20, 22–24, 26] were rated at high risk in five trials. Selection bias [18–28, 23–25] and selective reporting bias [18–19, 23–26] (significant or imbalanced missing outcome data) were rated at high risk in six trials. Other biases (RCTs with no statements on sources of funding/conflicts of interest) were rated at high risk in three trials [24–26]. Overall, all eight included RCTs were rated at high risk of bias.

### GRADE Evidence Profile (EP) and Summary of Findings (SoF)

The EP (Table 3) displays a detailed quality assessment and includes a judgment of each factor that determined the quality of evidence for each outcome. The SoF tables (Tables 4–6) include an assessment of the quality of evidence for each outcome.

**Table 3:** GRADE evidence profile: Intervention vs no intervention/control.

Quality Assessment						Summary of Findings			
Outcome (No. of studies; design)	Limitations	Inconsistency	Indirectness	Imprecision	Publication Bias	Intervention	No Intervention/ Control	SMD (95% CI)	Quality
HFS Knowledge up to 4 months (2 RCTs)	Serious limitations	No serious inconsistency	No serious indirectness	Serious imprecisions	Likely	278/535	257/535	SMD 0.38 (0.21–0.55)	⊕⊝⊝⊝ very low
HFS Behaviour up to 4 months (2 RCTs)	Serious limitations	Serious inconsistency	No serious indirectness	No Serious imprecisions	Likely	318/609	291/609	SMD 0.34 (-0.21–0.89)	⊕⊝⊝⊝ very low
HFS Knowledge at 2 months (1 RCT)	Serious limitations	No serious inconsistency	No serious indirectness	Very serious impression	Likely	49/96	47/96	SMD 0.66 (0.25–1.07)	⊕⊝⊝⊝ very low
HFS Behaviour at 6 months (1 RCT)	Serious limitations	No serious inconsistency	No serious indirectness	Very serious impression	Likely	91/277	186/277	SMD 0.35 (0.09–0.60)	⊕⊝⊝⊝ very low
HFS Behaviour at 12 months (1 RCT)	Serious limitations	No serious inconsistency	No serious indirectness	Very serious impression	Likely	91/277	186/277	SMD 0.36 (0.11–0.61)	⊕⊝⊝⊝ very low
HFS Knowledge Immediate (1 RCT)	Serious limitations	No serious inconsistency	No serious indirectness	Very serious impression	Likely	37/68	31/68	SMD -0.02 (-0.50–0.45)	⊕⊝⊝⊝ very low
HFS Behaviour Immediate (1 RCT)	Serious limitations	No serious inconsistency	No serious indirectness	Very serious impression	Likely	37/68	31/68	SMD 0.06 (-0.41–0.54)	⊕⊝⊝⊝ very low

**Table 4:** Summary of findings. Intervention vs No Intervention.

**Population:** primary school children. **Settings:** school setting. **Intervention:** Risk watch & Great escape. **Comparison:** No intervention. **Follow up:** up to 4 months.			
**Outcomes**	**SMD** (95% C.I.)	**No of participants** **(studies)**	**Quality of the evidence (GRADE)**
**HFS Knowledge:** (0 to 100). Higher values indicate better knowledge	SMD 0.38 (0.21–0.55)	535 (2 RCTs)	⊕⊝⊝⊝ very low^1^^,^^2^^,^^3^
**HFS Behaviour:** (0 to 100). Higher values indicate better behaviour	SMD 0.34 (-0.21–0.89)	609 (2 RCTs)	⊕⊝⊝⊝ very low^1^^,^^3^^,^^4^

Abbreviations: HFS; home fire safety, SMD; standardized mean difference, CI; confidence interval.

^1^We downgraded by one level due to high risk of bias.

^2^We downgraded by one level due to a relatively small sample size.

^3^We downgraded by one level due to publication bias.

^4^We downgraded by one level due to inconsistency.

**Table 5:** Summary of findings. Intervention vs Control.

**Population:** families with children. **Settings:** school, home and church. **Intervention:** counselling through verbal review & information **Comparison:** no intervention/limited information **Follow up:** 3 to 12 months.			
**Outcomes**	**SMD** (95% C.I.)	**No of participants** **(studies)**	**Quality of the evidence (GRADE)**
**HFS Knowledge:** (0 to 100). Higher values indicate better knowledge 3 months	SMD 0.66 (0.25–1.07)	96 (1 RCTs)	⊕⊝⊝⊝ very low^1^^,^^2^^,^^3^
**HFS Behaviour:** (0 to 100). Higher values indicate better behaviour 6 months	SMD 0.35 (0.09–0.60)	277 (1 RCTs)	⊕⊝⊝⊝ very low^1^^,^^2^^,^^3^
**HFS Behaviour:** (0 to 100). Higher values indicate better behaviour 12 months	SMD 0.36 (0.11–0.61)	277 (1 RCTs)	⊕⊝⊝⊝ very low^1^^,^^2^^,^^3^

Abbreviations: HFS; home fire safety, SMD; standardized mean difference, CI; confidence interval.

^1^We downgraded by one level due to high risk of bias.

^2^We downgraded by one level due to a relatively small sample size.

^3^We downgraded by one level due to publication bias.

**Table 6:** Summary of findings. Computer-based vs Instructor-based.

**Population:** adults **Settings:** hospital **Intervention:** computer-based fire safety intervention **Comparison:** instructor led fire safety intervention **Follow up:** Immediate			
**Outcomes**	**SMD** (95% C.I.)	**No of participants** **(studies)**	**Quality of the evidence (GRADE)**
**HFS Knowledge:** (0 to 100). Higher values indicate better knowledge	SMD -0.02 (-0.50–0.45)	68 (1 RCTs)	⊕⊝⊝⊝ very low^1^^,^^2^^,^^3^
**HFS Behaviour:** (0 to 100). Higher values indicate better behaviour	SMD 0.06 (-0.41–0.54)	68 (1 RCTs)	⊕⊝⊝⊝ very low^1^^,^^2^^,^^3^

Abbreviations: HFS; home fire safety, SMD; standardized mean difference, CI; confidence interval.

^1^We downgraded by one level due to high risk of bias.

^2^We downgraded by one level due to a relatively small sample size.

^3^We downgraded by one level due to publication bias.

### Participants

Among the eligible RCTs, four recruited parents/caregivers of children [20–23,26], three included primary school children [18–19, 24], and one recruited adult participants [25]. Among the eligible prospective cohort studies, one included school children [21], and the one recruited adult participant [27].

### Outcomes

Home fire safety knowledge was assessed in 7 RCTs and 2 prospective cohort studies [18–19, 22–26]. Home fire safety behaviour was examined in 6 RCTs [18–20, 22–23, 25]. The follow-up period ranged from immediate to 12 months post-intervention.

### Effects of intervention vs no intervention in primary school children (RCTs)

#### Home fire safety knowledge

**Fig 3:**
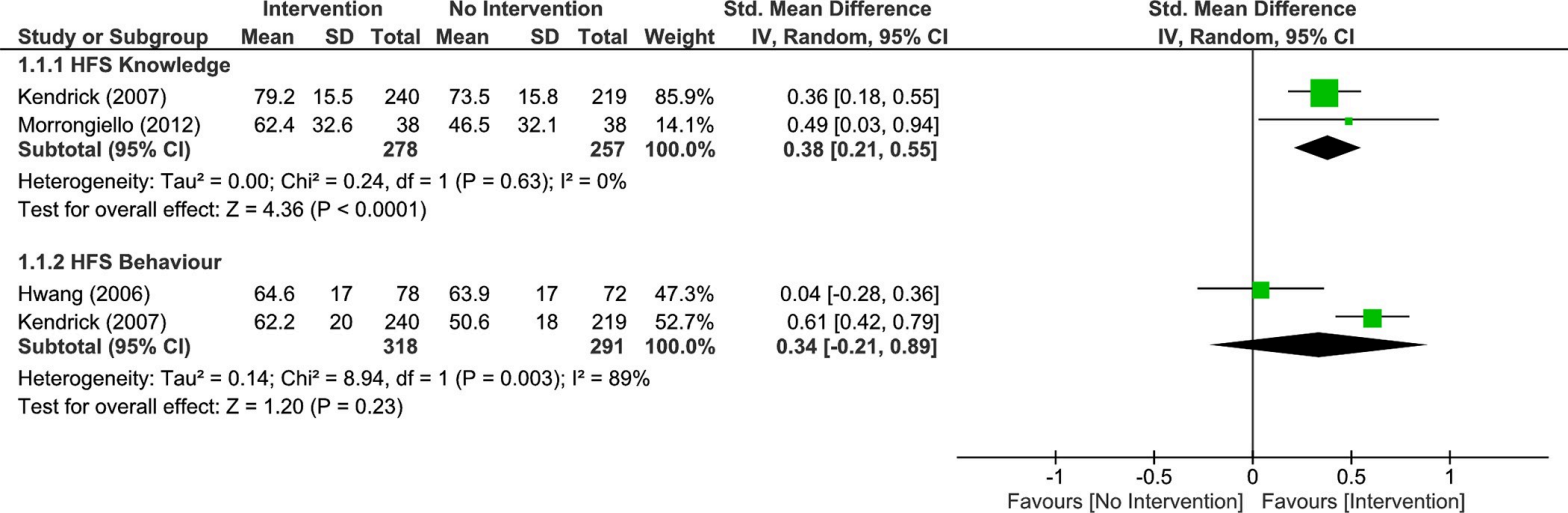
Analysis 1.1.1 forest plot of comparison: Intervention vs no intervention, up to 4 months–primary school children, outcome: Home fire safety knowledge, 2 RCTs. Analysis 1.1.2 Forest plot of comparison: Intervention vs No Intervention, up to 4 months–Primary School Children, outcome: Home Fire Safety Behaviour, 2 RCTs. Higher values indicate better/improved outcome.

Two studies were pooled to examine the effects of interventions (Risk Watch and Great Escape) vs no interventions on home fire safety knowledge at short-term (up to 4 months) follow up. The pooled results, demonstrated significant difference between groups (very low quality, 2 RCTs, 535 participants, standardized mean difference (SMD) 0.38, 95% CI: 0.21 to 0.55, p < 0.001, Fig 3; Analysis 1.1.1). Heterogeneity was absent. Given that an MCID is approximately 0.5 SD, the pooled results were not clinically important. However, more data are required to make a definitive conclusion.

#### Home fire safety behaviour

Two studies were pooled to assess the effects of interventions (Risk Watch and Great Escape) vs no interventions on home fire safety behaviour at short-term (up to 4 months) follow up. The pooled results, displayed no significant difference between groups (very low quality, 2 RCTs, 609 participants, SMD 0.34, 95% CI: -0.21 to 0.89, p = 0.23, Fig 3; Analysis 1.1.2). Heterogeneity was high, and we were not powered to conduct sub-group analysis. Given the MCID of 0.5 SD, the pooled results were not clinically important. However, more data are required to make a definitive conclusion.

### Effects of Intervention vs no intervention in primary school children (prospective cohort)

#### Home fire safety knowledge

**Fig 4:**
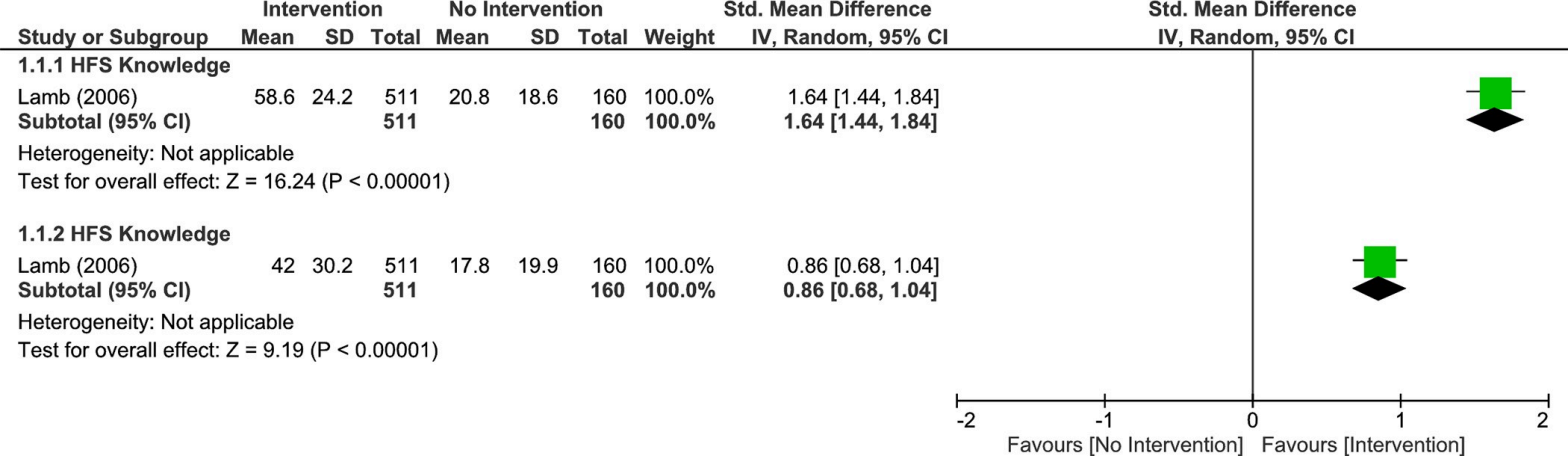
Analysis 4.1.1 forest plot of comparison: Intervention vs no intervention, immediate–primary school children, outcome: Home fire safety knowledge, 1 study. Analysis 4.1.2 Forest plot of comparison: Intervention vs No Intervention, 3 months–Primary School Children, outcome: Home Fire Safety Knowledge, 1 study. Higher values indicate better/improved outcome.

One study examined the effects of intervention (Life-skill protocol) vs no interventions on home fire safety knowledge immediately post-intervention. The results, displayed significant difference between groups (1 study, 671 participants, SMD 1.64, 95% CI: 1.44 to 1.84, p < 0.001, Fig 4; Analysis 1.1.1). We found similar results at short-term (3 months) follow up (1 study, 671 participants, SMD 0.86, 95% CI: 0.68 to 1.04, p < 0.001, Fig 4; Analysis 1.1.2). Given that an MCID is approximately 0.5 SD, the results were clinically important. However, this was a prospective cohort study, and we were unable to make a definitive conclusion on the effectiveness of the Life-skill protocol in improving home fire safety knowledge.

### Effects of intervention vs control in families with children (RCT)

#### Home fire safety knowledge

**Fig 5:**
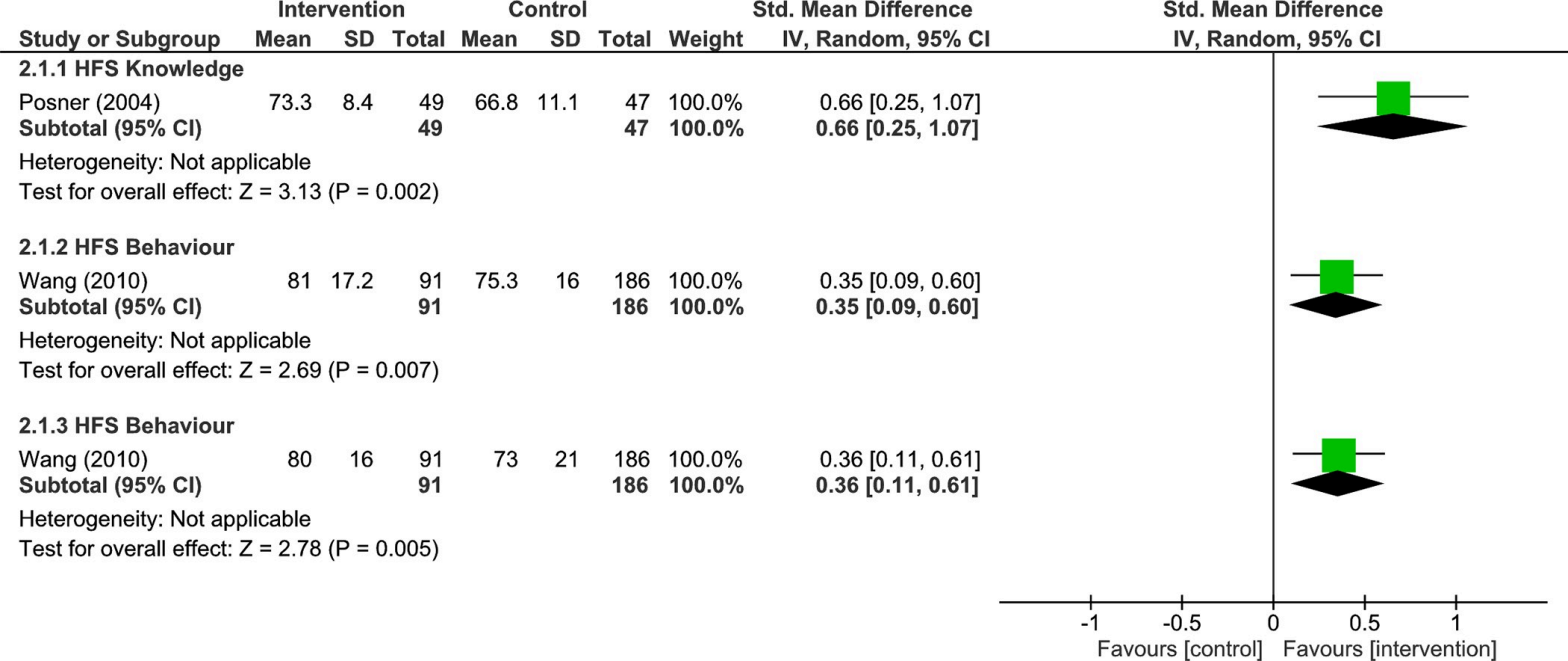
Analysis 2.1.1 forest plot of comparison: Intervention vs control, 2 months–families with children, outcome: Home fire safety knowledge, 1 rct. Analysis 2.1.2 Forest plot of comparison: Intervention vs Control, 6 months–Families with Children, outcome: Home Fire Safety Behaviour, 1 RCT. Analysis 2.1.3 Forest plot of comparison: Intervention vs Control, 12 months–Families with Children, outcome: Home Fire Safety Behaviour, 1 RCT. Higher values indicate better/improved outcome.

One study assessed the effects of home fire safety intervention vs control (minimal intervention) on home fire safety knowledge at short-term (2 months) follow up. The results, displayed significant difference between groups (very low quality, 1 RCT, 96 participants, SMD 0.66, 95% CI: 0.25 to 1.07, p = 0.002, Fig 5; Analysis 2.1.1). Given that an MCID is approximately 0.5 SD, the results were clinically important. However, more data are required to make a definitive conclusion.

#### Home fire safety behaviour

One study examined the effects of home fire safety intervention vs control (minimal intervention) on home fire safety behaviour at intermediate-term (6 months) follow up. The results demonstrated significant difference between groups (very low quality, 1 RCT, 277 participants, SMD 0.35, 95% CI: 0.09 to 0.60, p = 0.007, Fig 5; Analysis 2.1.2). We found similar results at long-term (12 months) follow up, (very low quality, 1 RCTs, 277 participants, SMD 0.36, 95% CI: 0.11 to 0.61, p = 0.005, Fig 5; Analysis 2.1.3). Given the MCID of 0.5 SD, the results were not clinically important. However, more data are required to make a definitive conclusion.

### Effects of different modes of intervention in adults (RCT)

#### Home fire safety knowledge

**Fig 6:**
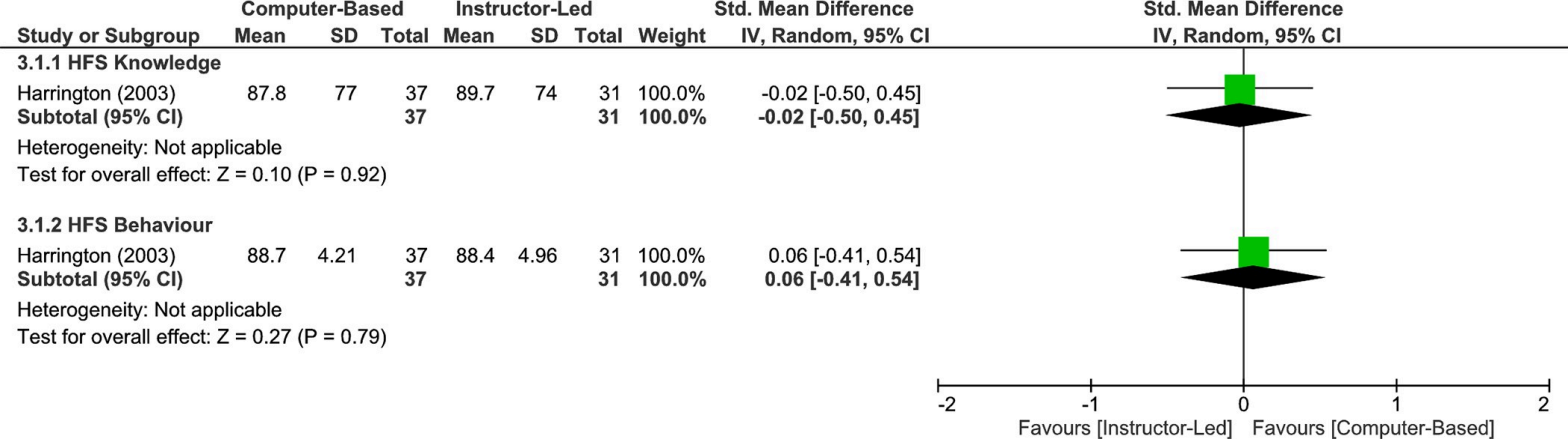
Analysis 3.1.1 Forest plot of comparison: Computer-based vs instructor-led, immediate–adults, outcome: Home fire safety knowledge, 1 RCT. Analysis 3.1.2 Forest plot of comparison: Computer-based vs Instructor-led, Immediate–Adults, outcome: Home Fire Safety Behaviour, 1 RCT. Higher values indicate better/improved outcome.

One study assessed the effects of different modes of home fire safety interventions (computer-based vs instructor-led) on home fire safety knowledge immediately post-intervention. The results, displayed no significant difference between groups (very low quality, 1 RCT, 68 participants, SMD -0.02, 95% CI: -0.50 to 0.45, p = 0.92, Fig 6; Analysis 3.1.1). Given that an MCID is approximately 0.5 SD, the results were not clinically important. However, more data are required to make a definitive conclusion.

#### Home fire safety behaviour

One study assessed the effects of different modes of home fire safety interventions (computer-based vs instructor-led) on home fire safety behaviour immediately post-intervention. The results, displayed no significant difference between groups (very low quality, 1 RCT, 68 participants, SMD 0.06, 95% CI: -0.41 to 0.54, p = 0.79, Fig 6; Analysis 3.1.2). Given that an MCID is approximately 0.5 SD, the results were not clinically important. However, more data are required to make a definitive conclusion.

## Discussions

This review identified and synthesized the most rigorously designed intervention studies, finding that there is a small number of studies examining diverse HFS interventions on knowledge and behaviour. In fire prevention research a major challenge is how researchers can ascertain whether a fire was prevented. Hence, they rely on test of knowledge of fire prevention strategies. The limitation, which is substantial, is that this may not insure these strategies are implemented. However, promising results were found in the small pool of studies in that statistically and clinically important improvements in HFS knowledge were found when different interventions were compared to the control or no intervention groups, in primary school children and families with children at up to 4 months follow up. We also found that there was no immediate difference in HFS knowledge and behavioural improvements between two ways of delivering HFS programs (instructor-led vs. computer-based).

Warda et al. (1999) review concluded that there is a need for intensive program evaluation, especially among school children demographic. In our review, we identified 3 RCTs and 1 prospective cohort study that examined the effectiveness of HFS interventions in this population. However, the magnitudes of intervention effects were different between the two study designs. In the Lamb et al. (2006) prospective cohort study (interventions vs no intervention groups), SMDs of 1.64 (95% CI: 1.44–1.84) and 0.86 (95% CI: 0.68–1.04) were reported for improvements in HFS knowledge and behaviour, respectively. These values were much higher than those reported in the 3 included RCTs (Kendrick et al. 2007; Morrongiello et al. 2012; Hwang 2006). It is likely that the magnitude of intervention effects was over-estimated by Lamb et al. (2006).

All eight trials identified in this review were rated at high risk of bias. The rating of very low-quality evidence per outcome across trials was based on the judgement of serious limitations (risk of bias), very serious imprecision and likely publication bias in all the outcomes across trials. This can be challenging area to conduct RCTs, and it likely that cluster-randomized trials may be needed to evaluate group interventions on a larger scale. Given that multiple approaches are likely to reach and benefit different target audiences, it will require a much larger pool of studies to define the optimal approaches. Despite the limitations in current research, it is reassuring that the methods evaluated have had positive effects on knowledge, and this suggests that the methods that are currently being used at least have a positive effect on this precursor to behaviour change.

### Limitations

We focused on RCTs and prospective cohort studies and did not include conference papers, posters or abstracts. Therefore, there might be a source of publication bias within our search strategy.

## Conclusions

The limited evidence supports the use of HFS interventions to improve HFS knowledge and behaviour in children, families with children and adults. Large-scale well-designed randomized controlled trials that consider the unique nature of prevention research and look at behavioural or fire rates as outcomes in larger scale implementation are needed to further assess the effectiveness of HFS interventions.

## What is already known on this subject

- Residential fires remain a major public health burden
- There is a need to evaluate the effectiveness of Home Fire safety (HFS) programs among adults and especially among school-based education programs.

## What this study adds

- The state of the research is currently poor quality. Even randomized controlled studies are found to be poor quality. Future studies should be designed to remedy these flaws.
- Statistically and clinically important improvements in HFS knowledge were found between different interventions vs control or no intervention groups, in primary school children and families with children at up to 4 months follow up.
- No immediate differences were found in HFS knowledge and behavioral improvements between instructor-led vs. computer-based programs.

## Supporting information

S1 Checklist PRISMA 2009 checklist. (PDF)

S1 File Search strategy. (DOCX)
