# Supplementary material for: Effectiveness of home fire safety interventions. A systematic review and meta-analysis
Source: PLoS One. 2019 May 20;14(5):e0215724. doi: 10.1371/journal.pone.0215724 (PMC6527231; doi:10.1371/journal.pone.0215724)
Supplement: S1 File — (DOCX) [file pone.0215724.s002.docx]

| Appendix – 1 | | |
| --- | --- | --- |
| Search strategy. | | |
| Database | Search conditions |  |
| EMBASE | #1 home fire safety  #2 home fire safety knowledge  #3 home fire safety behaviour  #4 effectiveness of home fire safety  #5 residential fires  #6 Fire prevention programs  #7 Fire prevention programs adults  #8 Fire prevention programs children  #9 Fire prevention  #10 #1 AND #9  #11 #2 AND #9  #12 #3 AND #9  #13 #4 AND #9  #14 #5 AND #9 |  |

| Appendix – 1 | | |
| --- | --- | --- |
| Search strategy. | | |
| Database | Search conditions |  |
| MEDLINE | #1 home fire safety  #2 home fire safety knowledge  #3 home fire safety behaviour  #4 effectiveness of home fire safety  #5 residential fires  #6 Fire prevention programs  #7 Fire prevention programs adults  #8 Fire prevention programs children  #9 Fire prevention  #10 #1 AND #9  #11 #2 AND #9  #12 #3 AND #9  #13 #4 AND #9  #14 #5 AND #9 |  |

| Appendix – 1 | | |
| --- | --- | --- |
| Search strategy. | | |
| Database | Search conditions |  |
| PubMed | #1 home fire safety  #2 home fire safety knowledge  #3 home fire safety behaviour  #4 effectiveness of home fire safety  #5 residential fires  #6 Fire prevention programs  #7 Fire prevention programs adults  #8 Fire prevention programs children  #9 Fire prevention  #10 #1 AND #9  #11 #2 AND #9  #12 #3 AND #9  #13 #4 AND #9  #14 #5 AND #9 |  |
